# Decreasing concentrations of carbonaceous aerosols in China from 2003 to 2013

**DOI:** 10.1038/s41598-021-84429-w

**Published:** 2021-03-05

**Authors:** Yan Cheng, Judith C. Chow, John G. Watson, Jiamao Zhou, Suixin Liu, Junji Cao

**Affiliations:** 1 School of Human Settlements and Civil Engineering, Xi’an Jiaotong University, Xi’an, China; 2 State Key Laboratory of Loess and Quaternary Geology, Institute of Earth Environment, Chinese Academy of Sciences, Xi’an, China; 3 Division of Atmospheric Sciences, Desert Research Institute, Reno, NV USA; 4 Institute of Atmospheric Physics, Chinese Academy of Sciences, Beijing, China

**Keywords:** Environmental sciences, Planetary science

## Abstract

Carbonaceous aerosols were characterized in 19 Chinese cities during winter and summer of 2013. Measurements of organic carbon (OC) and elemental carbon (EC) levels were compared with those from 14 corresponding cities sampled in 2003 to evaluate effects of emission changes over a decade. Average winter and summer OC and EC decreased by 32% and 17%, respectively, from 2003 to 2013, corresponding to nationwide emission control policies implemented since 2006. The extent of carbon reduction varied by season and by location. Larger reductions were found for secondary organic carbon (SOC, 49%) than primary organic carbon (POC, 25%). PM_2.5_ mass and total carbon concentrations were three to four times higher during winter than summer especially in the northern cities that use coal combustion for heating.

## Introduction

Carbonaceous aerosols, including organic carbon (OC) and elemental carbon (EC), are important for their adverse effects on visibility, climate, and human health^1–5^. OC is one of the largest constituents of suspended particulate matter (PM) mass and originates mostly from combustion processes. While EC derives from incomplete combustion, OC results from both direct emissions and atmospheric reactions of organic gases that form condensable compounds^6,7^. Carbonaceous aerosols influence the climate directly by scattering and absorbing incoming solar radiation, and indirectly by acting as cloud condensation nuclei and/or ice nuclei, which modify the microphysics, radiative properties, as well as lifetime and extent of clouds^8^. Light scattering by OC cools both the Earth’s surface and the atmosphere^9^, while light absorption by EC heats the atmosphere, possibly reducing cloud cover (termed the “semidirect effect”)^10^. Carbonaceous aerosol in the 0.1 to 1.0 µm size range can be transported and deposited in the human respiratory tract and is associated with cardiopulmonary mortality^4,5,11,12^. Positive associations of OC and EC with cardiovascular and respiratory mortality are found in urban environments^13^.

Concerns over climate and health effects have prompted efforts to reduce carbonaceous aerosols, including improvements for vehicle engines and exhaust-treatment technologies, better fuel quality, traffic management optimization, and implementation of stringent emission standards. Environmental protection policies prior to 2005 were not effective for reducing source emissions^14,15^. However, the 11th Five-Year Plan (2006–2010) set quantitative milestones for environmental and energy targets, including the “total emission control on SO_2_ [sulfur dioxide]” and an “energy saving” policy. These measures were maintained and extended in the 12th Five-Year Plan (2011–2015), which resulted in decreased ambient SO_2_ and PM_10_ emissions and concentrations^14^. Although a 10% reduction of SO_2_ and a 20% reduction of energy consumption per unit of gross domestic product (GDP) were reported by the end of the 11th Five-Year Plan (2006–2010)^14^, these reductions are somewhat offset by population and economic growth. From 2003 to 2013, fossil fuel consumption increased by 96.8%, with a 141% increase in vehicle-kilometers travelled (VKT)^16^. VKT increased faster in large cities due to rapid urbanization and motorization. The PM chemical composition has also changed over time due to changes in the emissions mixture. Increased flue gas desulfurization (FGD) applied to power and industrial sectors and the shift from gasoline to natural gas in some vehicle engine exhausts might increase emissions of volatile organic compounds (VOCs) and nitrogen oxides (NO_x_)^17–19^. Enhanced emissions from gasoline and liquefied petroleum gas (LPG) vehicles in Hong Kong were related to increases in VOC and ozone (O_3_) concentrations exceeding the ambient air quality objectives^20^.

Biases of 10–20% have been reported for different OC and EC measurements due to variations in sampler configuration, sampling sites, monitoring periods, and carbon analysis protocols^21–23^. To obtain long-term trends, comparable sampling and analysis methods are needed.

Nationwide measurements of PM_2.5_ mass (particles with aerodynamic diameter less than 2.5 µm), OC, and EC started in 14 Chinese cities during the summer and winter of 2003 to establish a baseline. This paper presents results from a follow-up study in 2013 for 19 cities, including the original 14. Comparison between 2003 and 2013 measurements are made to evaluate carbonaceous aerosol changes over the intervening decade.

## Method

### Sampling sites and descriptions

**Figure 1:**
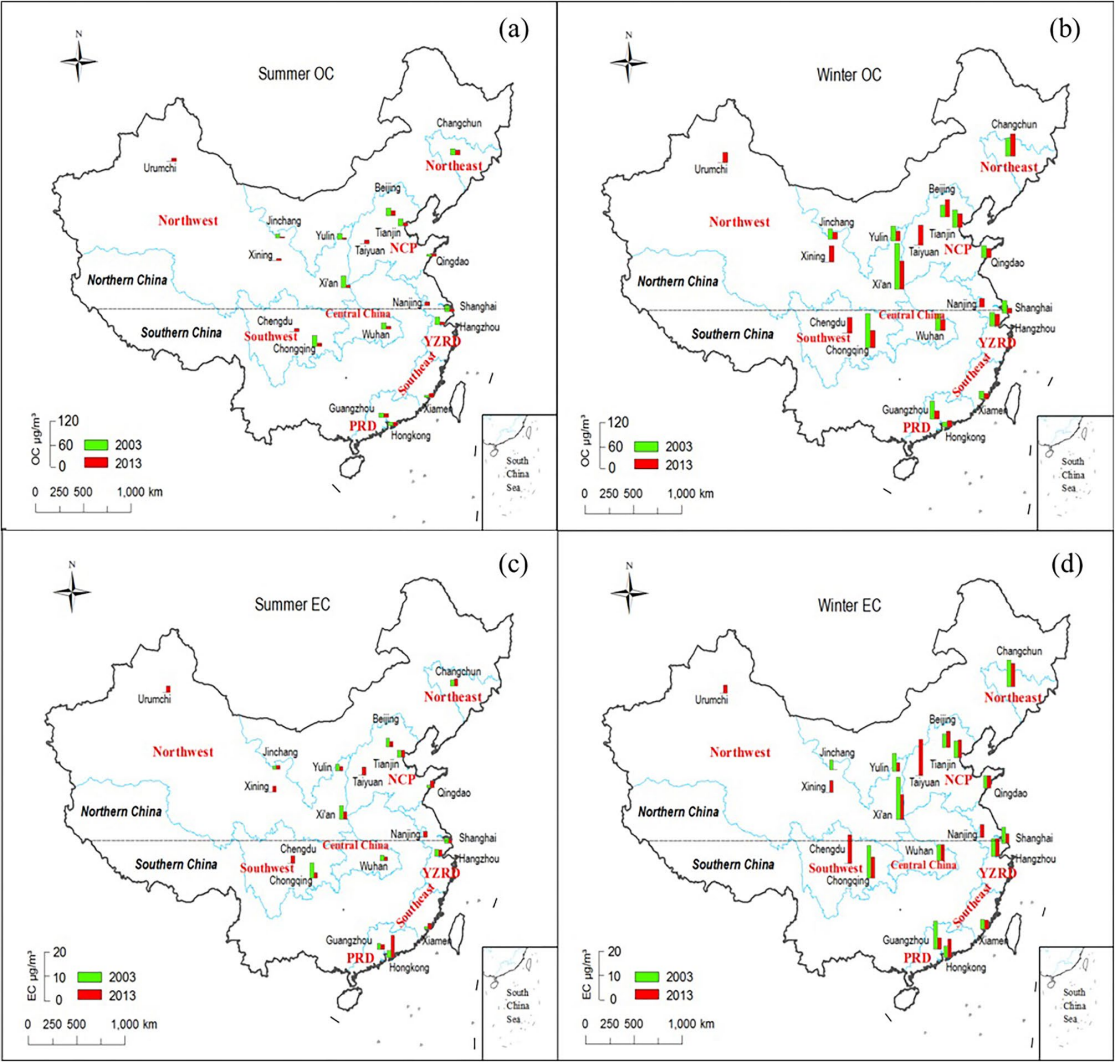
Comparisons of PM_2.5_ OC and EC between 2003 and 2013 indicated by bar height for: (**a**) summer OC, (**b**) winter OC, (**c**) summer EC, and (**d**) winter EC. The 2013 study includes ten cities in northern China (i.e., Changchun [CC], Urumqi [UR], Beijing [BJ], Tianjin [TJ], Jinchang [JC], Yulin [YL], Taiyuan [TY], Qingdao [QD], Xining [XN], and Xi’an [XA]) and nine cities in southern China (i.e., Nanjing [NJ], Shanghai [SH], Hangzhou [HZ], Wuhan [WH], Chengdu [CD], Chongqing [CQ], Xiamen [XM], Guangzhou [GZ], and Hong Kong [HK]).

Nineteen cities (Supplemental Table S1) including five megacities (i.e., Beijing [BJ], Tianjin [TJ], Shanghai [SH], Chengdu [CD], and Chongqing [CQ]) were selected to represent economically developed and developing urban regions, as shown in Fig. 1. The sampling network is documented in Table 1. In addition to those from the 2003 study^24^, three northern cities were added that represent developing industries (Taiyuan, TY), high altitudes (Xinning, XN), and arid western (Urumqi, UR) environments, along with two southern cities that represent developing urban environments (Chengdu, CD and Nanjing, NJ). Sampling sites represent urban-scale exposures, with most located at university campuses or research centers (> 100 m from local sources such as major roadways).

**Table 1:** Location and description of sampling sites in 19 cities for 2013 and 14 cities for 2003.

City name	Code	Location	Region description	Site type	Sampling site description
**Northern cities (n = 10)**					
Changchun, Jilin Province	CC	43.9° N, 125.3° E	Northeastern old industrial bases	An urban-commercial site in a continental and developed industrial city	Roof (6 m) of a building at Jilin University
Urumqi, Xinjiang Uygur Autonomous Region	UR^a^	43.5° N, 87.4° E	Northwest China	An urban-commercial site in a continental developing city, far from the ocean	Roof (10 m) of a building at the Institute of Desert Meteorology
Beijing Municipality	BJ	39.9° N, 116.4° E	Beijing–Tianjin–Hebei region	An urban-commercial site in the capital of China, megacity	Roof (14 m) of a building at Institute of Atmospheric Physics, Chinese Academy of Sciences (CAS)
Tianjin, Hebei Province	TJ	39.1° N, 117.2° E	Beijing–Tianjin–Hebei region	An urban site in a developed industrial city	Roof (20 m) of a building at Nankai University
Jinchang, Gansu Province	JC	383° N, 101.1° E	Surrounded by the Gobi Desert	An urban site in the Asian dust source region, non-urban desert	Roof (10 m) of a building at Jinchang Meteorological Bureau
Yulin, Shaanxi Province	YL	38.3° N, 109.8° E	The juncture of loess plateau and Maowusu desert	An suburban site in a continental developing city, close to a desert	A observation tower (10 m) in Shaanxi Desert Institute
Taiyuan, Shanxi Province	TY^a^	37.5° N, 112.3° E	Coal base	An urban-commercial site in a continental and developing industrial city	Roof (20 m) of a building at Taiyuan University of Technology
Qingdao, Shandong Province	QD	36° N, 120.3° E	Coastal region	An urban site in a developing coastal city	Roof (10 m) of a building at Chinese Ocean University
Xining, Qinghai Province	XN^a^	36.4° N, 101.5° E	The Northeastern Tibetan Plateau	A roadside-commercial site in a continental developing city, high altitude	Roof (18 m) of a building at Xining Environmental Monitoring Center
Xi’an, Shaanxi Province	XA	34.2° N, 108.9° E	Fen Wei Plain	An urban site in a continental and developing industrial city	Roof (10 m) of a building at Institute of Earth Environment, CAS
**Southern cities (n = 9)**					
Nanjing, Jiangsu Province	NJ^a^	32° N, 118.5° E	Yangtze River Delta region	An urban-commercial site in a developing city	Roof (80 m) of a building at Nanjing University
Shanghai Municipality	SH	31.2° N, 121.4° E	Yangtze River Delta region	An urban site in an industrial and commercial megacity	Roof (8 m) of a building at Donghua University
Hangzhou, Zhejiang Province	HZ	30.2° N, 120.1° E	Yangtze River Delta region	An urban site in an developing continental city	A substation (20 m) at Hangzhou Environmental Monitoring Station
Wuhan, Hubei Province	WH	30.5° N, 114.2° E	Jianghan Plain	An urban site in an industrial and commercial city	Roof (8 m) of a building at Chinese University of Geosciences
Chengdu, Sichuan Province	CD^a^	30.4° N, 104° E	Sichuan Basin	An urban-commercial site in a continental developing city	Roof (18 m) of a building at Chengdu Branch, Chinese Academy of Sciences
Chongqing Municipality	CQ	29.5° N, 106.5° E	Sichuan Basin	An urban-commercial site in a continental and developing industrial city	Roof (10 m) of the Chongqing Academy of Environmental Sciences
Xiamen, Fujian Province	XM	24.4° N, 118.1° E	Fen Wei Plain	An urban site in an coastal and commercial city, developing city	Roof (8 m) of a building at Xiamen University
Guangzhou, Guangdong Province	GZ	23.1° N, 113.2° E	Pearl River Delta	An urban site in an industrial and commercial megacity	Roof (10 m) of a building at Zhongshan Uinversity
Hong Kong Special Administrative Region	HK	22.2° N, 114.1° E	Pearl River Delta/Coastal region, southern China	A roadside-commercial site in a coastal and commercial city, developed city	A monitoring site (10 m) at Hong Kong Polytechnic University

^a^The five cities that are not included in the 2003 study^24^.

### Sample collection

Integrated daily, 24 h PM_2.5_ sampling (0900 to 0900 local standard time) was conducted during winter (5–26 January) and summer (1–31 July) of 2013 (Table S2). PM_2.5_ samples were collected on 836 prefired (900 °C for 3 h) 47 mm Whatman QMA quartz-fiber filters, using mini-vol air samplers (Airmetrics, Eugene, OR, USA) at a flow rate of 5 L min^−1^. These samplers were located on rooftops at varying heights (~ 6–20 m) above the ground level (Table 1). Quartz-fiber filters were analyzed gravimetrically for mass concentrations^25^ using a Sartorius MC5 electronic microbalance with a ± 1 μg sensitivity (Sartorius, Gottingen, Germany).

To minimize particle volatilization and aerosol liquid water biases, these filters were weighed after 24-h equilibration at a constant (within ± 2 °C) temperature between 20 and 23 °C and within ± 5% relative humidity (RH) between 30 and 40% following U.S. EPA guidelines^26^. Nominal values of 20 °C and 30% RH best conserve particle deposits during sample weighing^26^. Each filter was weighed at least twice before and after sampling to ensure reproducibility. The differences between the replicated weights were < 10 μg per filter for laboratory blanks and < 20 μg for exposed samples.

### Thermal/optical carbon analysis

A 0.5 cm^2^ punch from each quartz-fiber filters was analyzed for eight carbon fractions following the IMPROVE_A (Interagency Monitoring of Protected Visual Environments) thermal/optical reflectance (TOR) protocol^27–29^ using a DRI Model 2001 Carbon Analyzer (Atmoslytic Inc., Calabasas, CA). This protocol reports four OC fractions (OC1 to OC4 at 140 °C, 280 °C, 480 °C, and 580 °C, respectively, in a 100% helium [He] atmosphere); and three EC fractions (EC1 to EC3 at 580 °C, 740 °C, and 840 °C, respectively, in a 2% oxygen [O_2_]/98% He atmosphere). A laser beam at 633 nm monitored changes in optical reflectance and estimated pyrolyzed carbon (OP). IMPROVE_TOR OC is the sum of four OC fractions plus OP, whereas EC is the sum of three EC fractions minus OP^27^, with total carbon being the sum of OC and EC. Average field blanks were low, 0.97 and 0.09 µg cm^−2^ for OC and EC, respectively.

### Estimation of primary and secondary OC (POC and SOC)

While particulate EC is directly emitted from primary sources, particulate OC originates from both direct particulate emissions (primary OC, POC) and conversion of directly emitted gases to particles (secondary OC, SOC). POC includes directly emitted particulate OC and vapors condensed onto the particulate phase as the emissions cool^30^. SOC starts its atmospheric life in the gas phase as VOCs which undergo chemical transformations to less volatile compounds that shift to the particulate phase^30^. Although POC tends to dominate in most polluted areas, SOC can exceed POCs contribution during haze episodes. Turpin et al.^31^ reported ~ 80% of OC as SOC during photochemical smog event in southern California, USA. Huang et al.^32^ found 44–71% SOC in organic aerosols in four Chinese cities (i.e., Beijing, Shanghai, Xi’an, and Guangzhou).

Multiple methods have been reported to estimate SOC. Wu and Yu^33^ propose the use of the minimum R squared (MRS) method, as the MRS method presents a quantitative criterion to determine the primary OC and EC ratio ((OC/EC)_pri_), which minimizes the uncertainties in estimating SOC. In comparing five linear regression techniques, Wu and Yu^34^ further recommended the use of Deming regression, orthogonal distance regression, and York regression to estimate SOC.

As formation of SOC, often referred to secondary organic aerosol (SOA), involves complicated atmospheric processes of photochemical oxidation, gas/particle partitioning and nucleation/condensation, it presents a large challenge to accurately estimate SOC. There are pros and cons of applying different methods to estimate SOC with uncertainties associated with each. The underlying principle of the tracer approach^31^ is based on the assumption of a fixed relationship between primary OC and EC, while in reality, the OC and EC ratios and background concentrations vary over time. To ensure adequate validity of the (OC/EC)_pri_, data from extreme weather conditions (e.g., rain or storms) and from combustion dominated environment (with high OC/EC ratios) are excluded, and OC/EC measurements with the lowest potential in photochemical production (i.e., lowest 20% of OC/EC ratios) are retained to estimate (OC/EC)_pri_^24,35,36^. The EC tracer approach used in this study intends to maintain consistency in SOC comparison with the 2003 study.

Table S3 summarizes linear regressions between OC and EC, with similar findings for both years. During winter, good OC/EC correlations (R^2^: 0.66–0.95) were found for all but two northern industrial cities (i.e., Changchun and Xi’an), consistent with contributions from a mixture of sources (e.g., residential and commercial coal combustion, and motor vehicle exhaust). Correlations between OC and EC (R^2^: 0.16–0.92) were variable in summer, implying different source mixtures.

The OC/EC slopes were 2.34 and 2.08 in winter for the northern and southern cities, respectively, similar to those of 2.81 and 2.10 in 2003. As shown in Table S4, the summer OC/EC ratios of 0.97 and 0.51 during 2013 in the northern and southern cities are lower than the corresponding 2003 values of 1.99 and 1.29.

### Meteorological measurements

Table S5 summarizes average meteorological measurements by season for 2003 and 2013. Besides rainfall, cities in northern and southern China showed similar levels of atmospheric pressure, temperature, relative humidity (RH), and wind speed between 2003 and 2013 for both seasons. An increase in precipitation was found for both northern and southern cities during the summer of 2013. Summertime rainfall in Xiamen, Guangzhou, Chongqing, Beijing, and Yulin exceeded by twofold that in 2003. In contrast, precipitation decreased during the winter of 2013 in southern cities. There were over twofold higher rainfalls in 2003 for Xiamen, Guangzhou, Shanghai, Nanjing, and Chengdu. Average precipitation for northern cities were 6 and 4.5 mm for 2003 and 2013, respectively, generally lower than 10 mm per city.

## Results and discussion

### Seasonal variations of OC and EC in 2013

Table 2 summarizes average PM_2.5_ mass and carbon concentrations by season for each city and region. Average wintertime PM_2.5_ mass (167.6 ± 97.9 µg m^−3^) in the north were ~ 23% higher than in the south (135.7 ± 68.0 µg m^−3^). Wintertime PM_2.5_ mass concentrations were ~ 2.8–3 times higher than summer levels (43.8–60.9 µg m^−3^). Carbonaceous aerosol (1.6 × OC plus EC, with a factor of 1.6 to compensate for unmeasured oxygen and hydrogen^37^) accounted for 30–40% of the PM_2.5_ mass. Average northern OC concentrations were 34.7 ± 21.4 µg m^−3^ in winter, ~ 50% higher than for the southern region (22.9 ± 11.6 µg m^−3^), and five times those of the summer OC (6.8 ± 3.6 µg m^−3^). EC concentrations were over twice as high in winter (8.5 ± 5.7 µg m^−3^) than in summer (3.2 ± 1.7 µg m^−3^) with less regional variability.

**Table 2:** Concentrations of OC and EC among 19 cities in 2013.

Cities^a^	Winter									Summer								
	PM_2.5_^b^	TC	OC	EC	OC/EC	CM,^c^%	POC	SOC	N^d^	PM2.5^b^	TC	OC	EC	OC/EC	CM,%	POC	SOC	N^d^
**Northern cities**																		
CC	170.9 ± 62.8	61.4 ± 21.9	49.5 ± 20.4	11.9 ± 4.6	4.5 ± 2.1	54.3 ± 9.1	30.2 ± 10.7	20.0 ± 19.8	21	43.0 ± 18.1	13.2 ± 6.3	9.6 ± 4.2	3.6 ± 2.3	3.1 ± 1.2	53.4 ± 21.1	3.6 ± 2.3	6.0 ± 2.7	20
UR*	191.3 ± 76.8	27.7 ± 8.8	23.5 ± 7.3	4.3 ± 1.6	5.8 ± 1.3	23.9 ± 8.2	12.3 ± 3.8	11.1 ± 4.4	21	37.7 ± 9.5	10.8 ± 3.0	7.4 ± 2.0	3.3 ± 1.1	2.3 ± 0.4	42.1 ± 13.7	3.4 ± 1.0	4.0 ± 1.4	21
BJ	162.7 ± 111.2	48.2 ± 25.4	39.8 ± 21.1	8.4 ± 4.4	4.8 ± 0.5	51.0 ± 14.8	21.9 ± 10.4	17.9 ± 11.4	21	67.2 ± 36.3	13.4 ± 2.8	10.6 ± 2.2	2.8 ± 0.7	3.9 ± 0.6	36.1 ± 15.7	2.8 ± 0.7	7.7 ± 1.7	23
TJ	197.0 ± 98.7	39.1 ± 19.4	30.0 ± 15.6	9.1 ± 4.0	3.2 ± 0.5	29.2 ± 3.2	23.7 ± 9.4	6.5 ± 7.1	21	96.7 ± 37.6	10.8 ± 3.4	7.1 ± 2.5	3.7 ± 1.1	1.9 ± 0.5	18.5 ± 8.1	3.8 ± 1.1	3.3 ± 2.0	22
JC	87.2 ± 35.0	20.3 ± 7.5	15.9 ± 5.8	4.4 ± 1.7	3.7 ± 0.5	36.3 ± 12.3	12.6 ± 4.0	3.4 ± 2.4	22	31.6 ± 15.1	4.0 ± 2.2	2.2 ± 1.5	1.9 ± 0.7	1.1 ± 0.6	22.4 ± 26.0	1.9 ± 0.7	0.5 ± 0.7	20
YL	77.2 ± 34.1	26.8 ± 13.3	22.1 ± 11.2	4.7 ± 2.2	4.8 ± 0.8	52.6 ± 14.1	13.4 ± 5.2	8.7 ± 6.5	21	50.6 ± 37.4	6.5 ± 2.5	4.2 ± 1.5	2.3 ± 1.2	2.0 ± 0.6	22.9 ± 11.7	2.4 ± 1.1	1.9 ± 1.1	20
TY*	231.0 ± 102.2	64.0 ± 24.5	45.3 ± 18.7	18.6 ± 6.2	2.4 ± 0.4	41.3 ± 7.1	45.9 ± 14.5	2.9 ± 4.6	21	87.7 ± 31.5	13.3 ± 3.1	9.0 ± 2.1	4.3 ± 1.2	2.2 ± 0.3	23.4 ± 7.7	4.3 ± 1.1	4.7 ± 1.3	21
QD	134.4 ± 62.9	26.7 ± 12.1	20.3 ± 8.9	6.4 ± 3.2	3.3 ± 0.4	30.1 ± 5.4	17.2 ± 7.6	2.9 ± 2.4	21	50.6 ± 32.1	7.8 ± 3.6	5.8 ± 2.6	2.0 ± 1.1	3.1 ± 0.7	30.7 ± 23.5	2.1 ± 1.0	3.7 ± 1.7	14
XN*	156.1 ± 67.0	42.2 ± 17.4	36.2 ± 14.5	6.0 ± 3.1	6.3 ± 0.9	42.1 ± 13.6	16.5 ± 7.2	19.7 ± 8.2	22	52.4 ± 17.9	7.6 ± 3.8	4.6 ± 2.5	2.9 ± 1.4	1.6 ± 0.5	18.4 ± 5.9	3.0 ± 1.4	1.7 ± 1.4	21
XA	274.6 ± 113.2	75.2 ± 28.2	64.1 ± 25.5	11.2 ± 4.5	6.1 ± 2.3	42.6 ± 6.4	28.5 ± 10.5	35.7 ± 21.8	21	72.0 ± 47.1	10.5 ± 5.6	6.4 ± 3.5	4.1 ± 2.7	2.1 ± 1.2	24.3 ± 11.6	4.1 ± 2.6	2.6 ± 2.4	30
**Southern cities**																		
NJ*	143.3 ± 41.3	27.2 ± 7.5	20.5 ± 5.8	6.7 ± 1.8	3.1 ± 0.3	28.1 ± 5.2	15.9 ± 3.6	4.6 ± 2.7	21	36.6 ± 12.8	12.1 ± 3.8	9.0 ± 2.8	3.2 ± 1.1	2.9 ± 0.4	49.7 ± 12.0	4.3 ± 0.5	4.6 ± 2.4	21
SH	98.6 ± 41.1	15.9 ± 6.4	10.9 ± 4.6	5.0 ± 2.1	2.2 ± 0.5	23.9 ± 8.1	12.4 ± 4.3	0.5 ± 1.3	20	33.5 ± 17.2	8.8 ± 4.7	6.8 ± 3.9	2.0 ± 0.8	3.3 ± 0.8	38.3 ± 10.1	3.7 ± 0.4	3.3 ± 3.3	21
HZ	160.5 ± 44.3	35.6 ± 14.7	26.5 ± 11.2	9.1 ± 3.5	2.9 ± 0.3	31.5 ± 6.2	21.1 ± 7.3	5.6 ± 4.3	21	52.2 ± 14.2	10.5 ± 4.6	7.1 ± 3.8	3.4 ± 1.0	2.0 ± 0.7	27.3 ± 5.6	4.4 ± 0.5	2.7 ± 3.3	21
WH	184.1 ± 45.9	33.4 ± 7.5	24.9 ± 5.0	8.5 ± 2.8	3.1 ± 0.6	27.4 ± 7.1	19.7 ± 5.8	5.3 ± 3.2	21	44.2 ± 14.7	8.8 ± 2.5	6.7 ± 2.3	2.1 ± 0.5	3.4 ± 1.1	31.5 ± 11.1	3.7 ± 0.3	3.0 ± 2.0	21
CD*	230.6 ± 79.0	50.9 ± 12.1	36.0 ± 9.0	14.8 ± 3.4	2.4 ± 0.3	33.5 ± 9.7	32.9 ± 7.0	3.6 ± 3.6	21	69.4 ± 25.1	11.4 ± 6.5	7.5 ± 4.3	3.9 ± 2.2	1.8 ± 0.5	26.4 ± 11.4	4.7 ± 1.1	3.1 ± 2.9	22
CQ	172.6 ± 40.5	49.9 ± 11.4	38.9 ± 8.9	11.0 ± 2.8	3.6 ± 0.4	42.6 ± 2.8	24.9 ± 5.8	14.0 ± 4.7	21	52.0 ± 16.8	10.2 ± 2.5	7.3 ± 1.8	2.9 ± 0.8	2.6 ± 0.3	28.8 ± 4.3	4.2 ± 0.4	3.1 ± 1.5	21
XM	72.4 ± 21.2	16.8 ± 4.5	12.3 ± 3.2	4.5 ± 1.5	2.8 ± 0.4	34.8 ± 6.4	11.4 ± 3.0	1.2 ± 1.3	20	28.8 ± 19.4	10.7 ± 4.0	7.7 ± 2.8	3.0 ± 1.6	2.9 ± 0.9	63.9 ± 22.6	4.2 ± 0.8	3.5 ± 2.3	25
GZ	86.3 ± 24.1	25.5 ± 9.8	19.5 ± 7.4	6.0 ± 2.7	3.4 ± 0.7	43.0 ± 10.7	14.6 ± 5.5	4.9 ± 3.8	21	39.8 ± 19.7	11.9 ± 5.8	9.3 ± 5.1	2.6 ± 1.0	3.9 ± 1.5	45.1 ± 7.0	4.0 ± 0.5	5.3 ± 4.8	21
HK	71.5 ± 21.4	24.9 ± 7.4	15.3 ± 5.1	9.6 ± 2.5	1.6 ± 0.2	48.0 ± 5.7	22.0 ± 5.2	0.0 ± 0.0	22	40.0 ± 10.2	19.4 ± 4.8	7.9 ± 2.2	11.5 ± 2.8	0.7 ± 0.1	60.9 ± 12.7	8.5 ± 1.4	0.3 ± 0.7	21
**19 cities**																		
Ave North	167.6 ± 97.9	43.1 ± 25.8	34.7 ± 21.4	8.5 ± 5.7	4.5 ± 1.7	40.4 ± 14.1	22.2 ± 13.3	12.5 ± 15.2	212	60.9 ± 37.6	9.9 ± 4.9	6.8 ± 3.6	3.2 ± 1.7	2.3 ± 1.1	29.0 ± 18.2	3.2 ± 1.7	3.6 ± 2.8	212
Ave South	135.7 ± 68.0	31.3 ± 15.2	22.9 ± 11.6	8.4 ± 4.0	2.8 ± 0.7	34.9 ± 10.5	19.6 ± 8.3	3.3 ± 6.4	188	43.8 ± 20.4	11.4 ± 5.3	7.7 ± 3.4	3.7 ± 3.0	2.6 ± 1.2	41.3 ± 18.1	4.6 ± 1.5	3.1 ± 3.2	194
Ave^e^	152.5 ± 86.5	37.6 ± 22.2	29.1 ± 18.5	8.5 ± 5.0	3.7 ± 1.6	37.8 ± 12.8	21.0 ± 11.3	8.2 ± 12.7	400	52.6 ± 31.6	10.6 ± 5.1	7.2 ± 3.5	3.4 ± 2.4	2.5 ± 1.1	35.0 ± 19.2	3.9 ± 1.7	3.3 ± 3.0	406
**14 cities**																		
Ave North	157.2 ± 100.5	42.5 ± 26.9	34.5 ± 23.2	8.0 ± 4.5	4.3 ± 1.6	42.3 ± 14.1	21.1 ± 10.6	13.4 ± 16.6	148	61.3 ± 40.0	9.7 ± 5.2	6.7 ± 3.9	3.0 ± 1.9	2.5 ± 1.2	29.0 ± 19.7	3.1 ± 1.8	3.6 ± 3.1	149
Ave South	121.0 ± 57.8	29.1 ± 14.4	21.3 ± 11.4	7.7 ± 3.5	2.8 ± 0.8	36.1 ± 10.8	18.2 ± 7.2	3.2 ± 7.0	146	41.3 ± 18.0	11.3 ± 5.3	7.5 ± 3.3	3.7 ± 3.2	2.7 ± 1.3	42.2 ± 18.5	4.6 ± 1.6	2.9 ± 3.3	151
Ave^e^	139.3 ± 84.0	35.8 ± 22.6	28.0 ± 19.5	7.9 ± 4.0	3.6 ± 1.5	39.2 ± 13.0	19.6 ± 9.2	8.3 ± 13.8	294	51.2 ± 32.4	10.5 ± 5.3	7.1 ± 3.6	3.4 ± 2.7	2.6 ± 1.3	35.7 ± 20.2	3.8 ± 1.9	3.3 ± 3.2	300

^a^See site description in Table 1.

^b^Values represent average ± standard deviation in µg m^−3^.

^c^CM,carbonaceous matter = 1.6 × OC + EC.

^d^Numbers of samples.

^e^Ave, average for Northern and Southern Cities.

*The five sites that ware not included in the 2003 study^24^.

The 19-city average 24 h PM_2.5_ mass concentration was 103.3 ± 82.3 μg m^−3^ (Table 3). Daily PM_2.5_ varied by a factor of 78, ranging from 5.8 μg m^−3^ (Qingdao, summer) to 451.2 μg m^−3^ (Beijing, winter). Approximately ~ 81% and ~ 18% of PM_2.5_ mass measurements exceeded China’s 24 h air quality standard of 75 μg m^−3^ during winter and summer, respectively. On average, carbonaceous aerosols constituted 36.4 ± 16.3% of PM_2.5_.

**Table 3:** Average winter and summer concentrations for PM_2.5_ mass and carbonaceous aerosols in 2003 and 2013.

	Annual average concentration (µg m^−3^)			Decrease in percent* (%)
	2013		2003	
	19 cities	14 cities	14 cities^#^	14 cities
PM_2.5_	103.3 ± 82.3	95.6 ± 77.5	117.6	19
TC	24.1 ± 21.0	23.2 ± 20.7	32.7	29
OC	18.2 ± 17.2	17.5 ± 17.4	26.0	32
EC	5.9 ± 4.6	5.6 ± 4.1	6.8	17
POC	12.4 ± 11.8	11.7 ± 10.3	15.6	25
SOC	5.8 ± 9.5	5.8 ± 10.3	11.3	49
OC/EC	3.1 ± 1.5	3.1 ± 1.4	4.0	23
CM,%	36.4 ± 16.3	37.5 ± 17.0	41.5	10

*(PM_2.5 2003_ − PM_2.5 2013_) × 100/PM_2.5 2003_.

^#^Winter and summer average in Table 2 of the 2003 study^24^.

**Figure 2:**
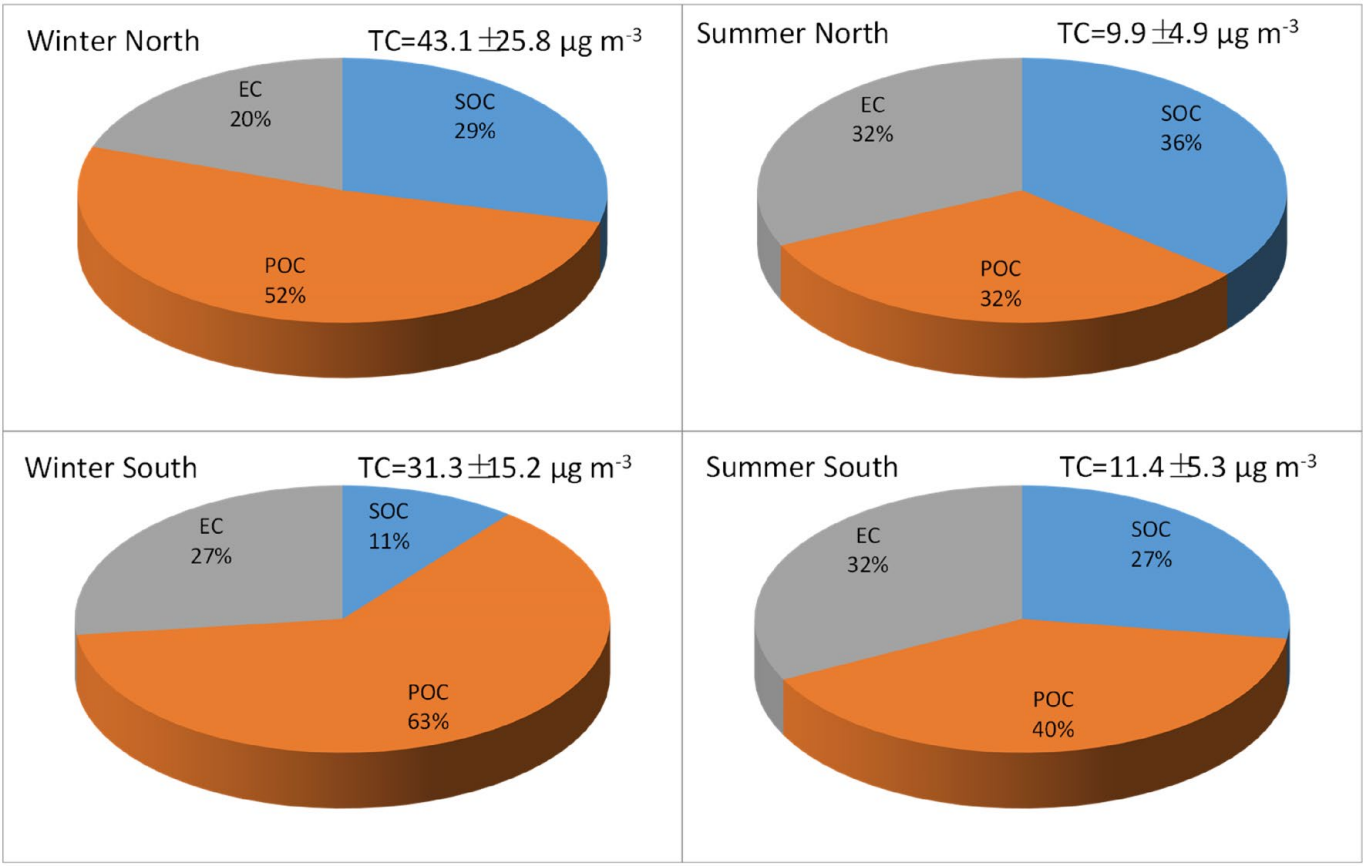
Percent of primary and secondary organic carbon (POC and SOC) and elemental carbon (EC) in total carbon (TC) for 19 cities in 2013.

Average northern POC and SOC concentrations were 22.2 ± 13.3 µg m^−3^ and 12.5 ± 15.2 µg m^−3^ in winter, accounting for ~ 52% and ~ 29% of TC, respectively (Fig. 2). Summer concentrations were lower (3.2–3.6 µg m^−3^), accounting for ~ 32% POC and ~ 36% SOC in TC. Winter is the most polluted season in northern China, with ~ 64% POC and ~ 36% SOC in OC; while summer is relatively clean, with ~ 47% POC and ~ 53% SOC.

In southern China, average wintertime POC concentrations were higher (19.6 ± 8.3 µg m^−3^, ~ 63% of TC) than those in summer (4.6 ± 1.5 µg m^−3^, ~ 40% of TC), with ~ 86% of OC as POC and ~ 14% as SOC. Minimal differences were found for average SOC between winter (3.3 ± 6.4 µg m^−3^, ~ 11% of TC) and summer (3.1 ± 3.2 µg m^−3^, ~ 27% of TC).

### Reduction in carbonaceous concentrations and implementation of control policies for 14 cities

For the 14 cities with 2003 results, Table 3 shows that average PM_2.5_ mass decreased by 19% from 117.6 µg m^−3^ in 2003 to 95.6 µg m^−3^ in 2013. A similar 19% reduction was found for EC, from 6.8 to 5.6 µg m^−3^; average OC decreased by 32% from 26.0 µg m^−3^ to 17.5 µg m^−3^. These reductions correspond to the emission control and energy policies of the 2006–2010 Five-Year Plan, despite increases of ~ 93% for coal consumption, ~ 77% for the crude oil usage, and ~ 470% for the number of motor vehicles^16^.

Annual average concentrations were 11.7 ± 10.3 µg m^−3^ for POC and 5.8 ± 10.3 µg m^−3^ for SOC in 2013, yielding ~ 25% and ~ 49% reductions, respectively, since 2003 (Table 3). POC reductions were more pronounced in summer (~ 61% in the north and ~ 26% in the south) than the corresponding ~ 28% and ~ 22% in winter. Summer SOC decreased by ~ 44% and ~ 58% for northern and southern China, respectively. Wintertime SOC experienced a ~ 73% reduction in southern China, but a ~ 10% increase was observed for northern China from 2003 to 2013, possibly due to less stringent VOC and NO_x_ controls for small boilers^14,19^. Past studies found that VOCs and NO_x_ emissions influence winter air quality in China^32,38^.

Average 14-city carbonaceous aerosols constituted ~ 38% and 42% of PM_2.5_ in 2003 and 2013, respectively. From 2013 to 2003, SOC and POC decreased by ~ 49% and 25%, respectively, whereas EC decreased by ~ 17%.

### 2013/2003 ratios for PM_2.5_ mass, OC, and EC

**Figure 3:**
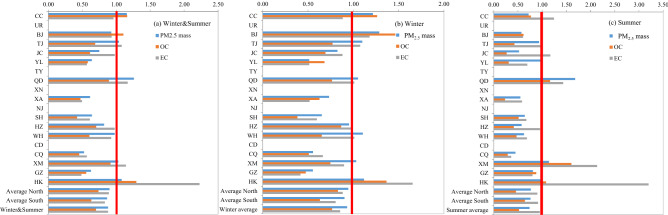
Ratios of 2013 to 2003 measurements for PM_2.5_ mass, OC, and EC concentrations in 14 cities (See Table 1 for site descriptions). No data available for Urumqui [UR], Taiyuan [TY], Xining [XN], Nanjing [NJ], and Chengdu [CD] in the 2003 study^24^.

Figure 3 illustrates nationwide pollution reduction, with most of the average 2013/2003 ratios less than unity for PM_2.5_ mass, OC, and EC. Concentration reductions are most apparent in the three northern (i.e., Jinchang, Xi’an, Yulin) and five southern (i.e., Chongqing, Guangzhou, Hangzhou, Shanghai, and Wuhan) cities. However, increased wintertime OC concentrations were found in Beijing and Changchun, whereas increased EC levels were found during winter in Tianjin and during summer in Qingdao and Xiamen. Hong Kong showed increases in OC and EC from 2003 to 2013 for both seasons.

### Changes of OC and EC concentrations between 2013 and 2003

Figure 1 shows that wintertime carbon decreased for most cities since 2003^24^. The most apparent wintertime OC reductions (~ 49–62%) were found for Shanghai (~ 62%), Guangzhou (~ 53%), and Chongqing (~ 49%); EC also decreased (34–58%) with ~ 34% for Chongqing, ~ 40% for Shanghai, and ~ 58% for Guangzhou. Reduction of wintertime carbonaceous aerosols for the remaining cities ranged from 13 to 37% for OC and 2–49% for EC. Shanghai, Guangzhou, and Chongqing represent the three most developed regions in southern China: the Yangtze River Delta (YRD) region, the Pearl River Delta (PRD) region, and the Chong-Yu (CY) region, respectively. Control measures such as industrial plant closures and traffic controls during the 2010 (May to October) World Exposition in Shanghai and the 2010 (November) Asian Games in Guangzhou demonstrated the effectiveness of regional air quality management strategies that were implemented on larger scales.

Increases in wintertime OC (~ 46%) and EC (~ 18%) in Beijing and the OC increase (~ 26%) in Changchun may be associated with severe haze episodes under sluggish weather systems that occurred over central and eastern China in 2013. During the two January episodes (9th–15th and 25th–31st), maximum hourly PM_2.5_ mass concentrations in Beijing were 680 and 530 μg m^−3^, respectively^39^. During January 2013, PM_2.5_ in Beijing ranged 106–451 μg m^−3^, consisting for 35–55% of carbonaceous aerosol. Ji et al.^23^ also noted elevated carbon concentrations during winter of 2016/2017 in the Beijing–Tianjin–Hebei region, which were attributed to intense coal combustion for residential heating and a high frequency of unfavorable meteorological conditions.

Compared to 2003, summertime OC decreased by 53–77% in seven cities including: Xi’an (~ 77%), Jinchang (~ 73%), Chongqing (~ 71%), Yulin (~ 69%), Hangzhou (~ 59%), Tianjin (~ 57%), and Wuhan (~ 53%). Summertime EC reductions varied by city, with 64% in Chongqing and 20–40% in Beijing, Yulin, Xi’an, Shanghai, Wuhan, and Guangzhou. In addition to emission reductions, improved atmospheric dispersion in summer favored lower concentrations. Air quality in Beijing improved before and during the 2008 summer Olympics due to the temporary implementation of pollution control measures. However, air pollution levels surged after the Olympics, signifying the importance of implementing long-term control measures.

Increases in summertime OC and EC since 2003 were found in coastal cities such as Qingdao, Xiamen, and Hong Kong, with low TC concentrations (7.8–19.4 μg m^−3^). Coastal cities usually experience lower air pollution levels because of clean marine air intrusions. However, increases of primary emissions in Hong Kong may have resulted in the increase of OC and EC.

### Spatial distribution of carbonaceous aerosols in 2013 and its association with economic development

Winter carbon concentrations in both monitoring years were high in inland cities and low in coastal and background cities. Lower wintertime OC concentrations were found in the coastal cities of Shanghai (10.9 ± 4.6 µg m^−3^), Xiamen (12.3 ± 3.2 µg m^−3^), and Hong Kong (15.3 ± 5.1 µg m^−3^) and the non-urban desert city of Jinchang (15.9 ± 5.8 µg m^−3^). The eight highest inland OC cities were Xi’an (64.1 ± 25.5 µg m^−3^), Changchun (49.5 ± 20.4 µg m^−3^), Taiyuan (45.3 ± 18.7 µg m^−3^), Beijing (39.8 ± 21.1 µg m^−3^), Chongqing (38.9 ± 8.9 µg m^−3^), Xining (36.2 ± 14.5 µg m^−3^), Chengdu (36.0 ± 9.0 µg m^−3^), and Tianjin (30.0 ± 15.6 µg m^−3^), with concentrations exceeding the 19 cities winter average OC of 29.1 ± 18.5 µg m^−3^.

With the exception of the Chongqing and Chengdu (capital of Sichuan Province) megacities, elevated concentrations were found in northern China, reflecting the impacts from industries and central heating systems during cold winters with poor dispersion. After its establishment as a Municipality in 1997, Chongqing’s population increased to > 30 million by 2014^16^. Both cities are located in the Sichuan basin surrounded by mountains and have warmer climates with less winter heating. They suffer from severe acid rain from coal combustion, with carbonaceous aerosols accounting for 34–43% of PM_2.5_ mass during winter.

Most of the cities with elevated wintertime OC concentrations are associated with decades-long economic development. For example, Changchun, the capital of Jilin Province, is the largest industrial and transportation hub in northeast China, producing ~ 9% of the Chinese automobiles in 2009, and it experienced a ~ 26% increase in OC from 2003 to 2013; whereas Taiyuan, the capital of Shanxi Province, produces ~ 25% of China’s coal (designated as the Taiyuan Coal Transaction Center since 2012), and reported average winter TC levels of 64.0 ± 24.5 µg m^−3^.

The highest summer OC (10.6 ± 2.2 µg m^−3^) was found in Beijing, but it is less than the lowest winter OC concentrations for the 19 cities. The minimum summer OC average occurred in Jinchang (2.2 ± 1.5 µg m^−3^), a desert city in Gansu Province (bordering Inner Mongolia to the north with a population of 0.2 million) with few local industries.

There were eight inland cities that exceeded the 19-city EC averages of 8.5 ± 5.0 µg m^−3^ during winter, ranging 9.1 to 18.6 µg m^−3^, including: Hangzhou (9.1 ± 3.5 µg m^−3^), Tianjin (9.1 ± 4.0 µg m^−3^), Hong Kong (9.6 ± 2.5 µg m^−3^), Chongqing (11.0 ± 2.8 µg m^−3^), Xi’an (11.2 ± 4.5 µg m^−3^), Changchun (11.9 ± 4.6 µg m^−3^), Chengdu (14.8 ± 3.4 µg m^−3^), and Taiyuan (18.6 ± 6.2 µg m^−3^). With the exception of Hangzhou and Hong Kong, these are industrial cities with abundant coal combustion.

Hong Kong reported an increase of ~ 29% in OC and ~ 122% in EC since 2003. Vehicle engine exhaust, especially from diesel vehicles, is an important contributor, accounting for 20–51% of fine particles^40–42^.

### Variability of OC/EC ratios

**Figure 4:**
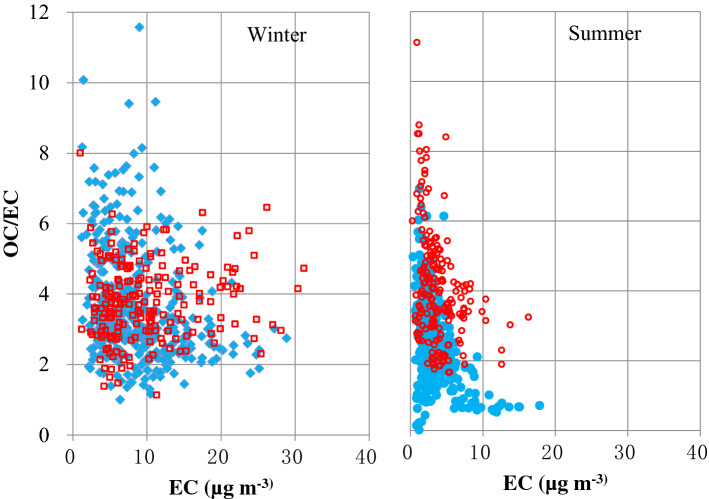
Distribution of OC/EC ratios versus EC concentrations in winter and summer of 2013 (filled blue diamonds) and 2003 (empty red circles).

Figure 4 shows variations in OC/EC ratios as a function of EC concentrations, ranging 1.2–11.6 during winter and 0.01–6.9 during summer of 2013. Similar variability was found for 2003 (red circles in Fig. 4) with lower OC/EC ratios in winter for concentrations < 20 µg m^−3^. Changes in PM carbon properties may influence the relative amounts of particle light scattering and absorption^24^ related to visibility impairment and climate change.

Higher winter OC/EC ratios (6.0 to 11.6) during 2013 (barely seen in 2003) were found in the northwestern cities of Urumqi, Yulin, Xining, and Xi’an. This may be due to changes in the source mixtures. Abundances of the thermal carbon fractions also changed. In wintertime Xi’an, high temperature carbon fractions of OC3 and OC4 (480 °C and 580 °C) were most abundant in 2013, whereas lower temperature OC2 (280 °C) and OC3 fractions dominated in 2003. These changes may reflect changes in the composition of organic compounds in the combustion emissions.

Ten northern cities (with the exception of Taiyuan) reported higher wintertime OC/EC ratios than southern cities. Taiyuan reported the lowest winter OC/EC ratio of 2.4, close to the ratio of 2.0 for primary fossil fuel combustion from the national emission inventory^43^. This is reasonable considering that Taiyuan is China’s coal capital, with coal energy, metallurgy, chemicals, and machinery industries^44^.

While the distribution pattern of OC/EC ratios during summer in 2013 was similar to that of 2003, the average ratio is 23% lower. This is attributed to the decrease in OC from 13.8 μg^.^m^−3^ in 2003 to 7.1 μg^.^m^−3^ in 2013 as the average EC concentrations (3.4–3.6 μg^.^m^−3^) remained similar. The summer OC/EC ratios were the highest in Beijing (3.0–4.8), followed by Guangzhou (1.9–6.9), with the lowest in Hong Kong (0.6–0.8). There are different vehicle fuel formulations among the cities. The higher proportion (~ 38%) of diesel vehicle emissions in Hong Kong resulted in lower OC/EC ratios. This is consistent with the average OC/EC ratio of 2.7 for gasoline and LPG vehicles and 0.5 for diesel vehicles^45^.

## Conclusions

Decreasing OC and EC concentrations were apparent at a national scale from 2003 to 2013; but the extent of reduction varied by season and location. Most inland cities showed decreases in carbon concentrations with the exception of Beijing and Changchun (capital of Jilin in northern China). Shanghai showed an apparent decrease, with a less distinct reduction in Qingdao and Xiamen for OC and slight increases for EC. The exception was found in Hong Kong, with a 2003 to 2013 increase of ~ 29% for OC and ~ 122% for EC.

Carbonaceous aerosols constituted ~ 37.5% and ~ 41.5% of PM_2.5_ mass for 2013 and 2003, respectively. Majority of the TC is in OC, with average OC/EC ratios of 3.1 and 4.0 for 2013 and 2003, respectively.

Summertime PM_2.5_ mass and OC concentrations were ~ 25–35% of those in winter with less variability in EC. Wintertime carbon concentrations were higher in northern than southern China, elevated in inland cities and low in coastal and non-urban desert cities. Winter in northern China is the most polluted season with ~ 64% primary organic carbon (POC) and ~ 36% of secondary organic carbon (SOC) in OC.

Wintertime OC/EC ratios were higher in most northern than southern cities, indicating the impact of haze episodes and fossil fuel combustion from heating. Elevated 2013 OC/EC ratios (6.0 to 11.6) in northwestern cities such as Urumqi, Yulin, Xining, and Xi’an, were barely seen in 2003. Changes in abundances among eight thermal carbon fractions suggest changes in emission properties over the past decade. Average summer 2013 OC/EC ratio of 2.5 was much lower than corresponding 2003 ratio of 4.2, with less variability in OC/EC ratios (3.7–3.8) during winter.

## Supplementary Information

Supplementary Information.
