# Supplementary material for: Decreasing concentrations of carbonaceous aerosols in China from 2003 to 2013
Source: Sci Rep. 2021 Mar 5;11:5352. doi: 10.1038/s41598-021-84429-w (PMC7935893; doi:10.1038/s41598-021-84429-w)
Supplement: Supplementary file 1 — Supplementary Information. [file 41598_2021_84429_MOESM1_ESM.docx]

**Decreasing concentrations of carbonaceous aerosols in China from 2003 to 2013**

Yan Cheng^1,3*^, Judith C. Chow^2,3^, John G. Watson^2,3^, Jiamao Zhou^3^, Suixin Liu^3^, Junji Cao^3,4*^

*^1^School of Human Settlements and Civil Engineering, Xi’an Jiaotong University, Xi’an, China*

*^2^Division of Atmospheric Sciences, Desert Research Institute, Reno, Nevada, USA*

*^3^State Key Laboratory of Loess and Quaternary Geology, Institute of Earth Environment, Chinese Academy of Sciences, Xi’an, China*

*^4^Institute of Atmospheric Physics, Chinese Academy of Sciences, Beijing, China*

Submitted to

Scientific Reports

November 24, 2020

*Corresponding authors:* [chengyan@xjtu.edu.cn](mailto:chengyan@xjtu.edu.cn)

[jjcao](mailto:cao@loess.llqg.ac.cn)@mail.iap.ac.cn

Table S1 Demographic information, vehicle numbers, and energy consumption data for 19 cities in 2013 and 14 cities in 2003.

Table S2 Sampling periods and number of samples from 2003 and 2013.

Table S3 Least-Squares Regression Results for OC and EC concentrations in 2003 and 2013.

Table S4 Least-Squares Regression Results for OC and EC Concentrations Grouped by OC/EC Ratio.

Table S5 Meteorological measurements during sampling periods in 2013 and 2003.
